# Supplementary material for: Association of Serum Vitamin B12 and Circulating Methylmalonic Acid Levels with All-Cause and Cardiovascular Disease Mortality among Individuals with Chronic Kidney Disease
Source: Nutrients. 2023 Jun 30;15(13):2980. doi: 10.3390/nu15132980 (PMC10343878; doi:10.3390/nu15132980)
Supplement: Supplementary file 1 [file nutrients-15-02980-s001.zip › nutrients-2429939-supplementary.pdf]

Supplementary Materials

Table S1. Baseline characteristics of study participants for CKD among U.S. adults by baseline serum vitamin B12 levels.

**Characteristic**	Participants, No. (%)					*P* value [a]
	Serum vitamin B_{12} levels, pmol/L					
	Total	Quartile 1 (<284.1)	Quartile 2 (284.1-397.1)	Quartile 3 (397.2-534.5)	Quartile 4 (≥534.6)	
**Participants, No.**	2589 (100.0)	593 (22.9)	677 (26.2)	636 (24.6)	683 (26.4)	-
**Age, years, mean (SE)**	59.04 (0.6)	57.69 (1.3)	57.87 (0.8)	58.57 (1.1)	62.02 (0.8)	0.016
**Sex, *n* (%)**						
Male	1324 (46.8)	326 (49.4)	377 (50.8)	313 (43.9)	308 (43.0)	0.034
Female	1265 (53.2)	267 (50.6)	300 (49.2)	323 (56.1)	375 (57.1)	
**Race, *n* (%)**						0.033
Hispanic	580 (12.6)	117 (11.1)	158 (14.4)	151 (12.9)	154 (11.9)	
Non-Hispanic white	1302 (69.7)	333 (75.5)	342 (68.8)	318 (69.6)	309 (65.2)	
Non-Hispanic black	543 (11.5)	107 (8.7)	139 (12.0)	128 (11.3)	169 (14.2)	
Others	164 (6.1)	36 (4.8)	38 (4.9)	39 (6.2)	51 (8.7)	
**Education, *n* (%)**						0.108
Less than high school	911 (25.2)	195 (21.5)	252 (29.7)	226 (23.1)	238 (26.2)	
High school	594 (24.2)	134 (23.7)	154 (22.5)	152 (26.0)	154 (24.6)	
College or higher	1084 (50.7)	264 (54.8)	271 (47.8)	258 (50.9)	291 (49.2)	
**Marital status, *n* (%)**						0.275
Married	1337 (53.9)	282 (49.5)	351 (53.9)	345 (55.9)	359 (56.2)	
Separated	980 (34.4)	238 (35.7)	255 (34.6)	219 (31.9)	268 (34.8)	
Never married	272 (11.9)	73 (14.8)	71 (11.5)	72 (12.1)	56 (9.1)	
**PIR, *n* (%)**						0.534
≤ 1	554 (17.0)	140 (18.5)	147 (18.3)	123 (15.8)	144 (15.5)	
1 to 4	1512 (54.9)	337 (53.6)	401 (53.9)	363 (52.9)	411 (59.1)	
≥ 4	523 (28.1)	116 (28.0)	129 (27.8)	150 (31.3)	128 (25.4)	
**Smoking, *n* (%)**						0.083
Never	2043 (76.9)	454 (74.1)	515 (73.8)	517 (79.6)	557 (80.0)	
Former	68 (3.1)	17 (4.4)	14 (2.0)	20 (3.9)	17 (2.3)	
Current	478 (20.0)	122 (21.5)	148 (24.3)	99 (16.5)	109 (17.7)	
**Drinking, *n* (%)**						0.378
None	900 (33.3)	181 (29.3)	236 (34.7)	231 (35.5)	252 (33.5)	
Moderate	1492 (58.2)	356 (60.3)	387 (55.9)	360 (56.3)	389 (60.2)	
Heavy	197 (8.6)	56 (10.4)	54 (9.4)	45 (8.2)	42 (6.3)	
**Physical activity, *n* (%)**						0.149
Low	1173 (49.1)	253 (47.6)	312 (48.4)	288 (49.8)	320 (50.5)	
Moderate	549 (21.3)	131 (23.3)	152 (23.2)	140 (22.5)	126 (16.3)	
High	867 (29.6)	209 (29.2)	213 (28.4)	208 (27.8)	237 (33.2)	
**CKD stages**						0.002
Stage 1	938 (41.1)	218 (44.4)	253 (44.1)	246 (40.0)	221 (35.8)	
Stage 2	668 (24.2)	157 (23.0)	178 (26.0)	168 (26.6)	165 (21.0)	
Stage 3	908 (32.8)	205 (31.6)	230 (28.0)	208 (32.1)	265 (39.4)	
Stage 4 and 5	75 (2.0)	13 (1.1)	16 (1.9)	14 (1.3)	32 (3.8)	
**BMI, kg/m², mean (SE)**	29.43 (0.2)	30.44 (0.4)	29.2 (0.4)	29.19 (0.4)	28.91 (0.4)	<0.001
**HEI-2015, mean (SE)**	53.08 (0.4)	51.05 (0.8)	51.84 (0.6)	54.11 (0.7)	55.32 (0.7)	<0.001
**Systolic blood pressure, mmHg, mean (SE)**	133.42 (0.5)	131.80 (1.3)	134.19 (1.0)	132.92 (1.2)	134.76 (1.2)	<0.001

eGFR, ml/min/1.73m², mean (SE)	81.42 (0.7)	82.39 (1.4)	83.92 (1.1)	82.32 (1.7)	77.04 (1.5)	<0.001
**MMA, nmol/L, mean (SE)**	217.13 (5.1)	302.68 (16.4)	207.16 (12.8)	175.95 (4.8)	183.86 (6.5)	<0.001

Abbreviations BMI, body mass index; CKD, chronic kidney disease; eGFR, estimated glomerular filtration rate; HEI-2015, Healthy Eating Index-2015; MMA, methylmalonic acid; PIR, family poverty-to-income ratio; SE, stand error; [a] *P* values were calculated using Rao-Scott Chi-square test for categorical variables and Wald F-test for continuous variables.

Table S2. Supplementary Table 2. Association of vitamin B_{12} levels with all-cause mortality and cardiovascular mortality among U.S. adults with CKD.

Cause of death	Vitamin B_{12} levels (pmol/L), HR (95% CI)					*P* for trend [a]
	Log (Vitamin B_{12})	Quartile 1 (<284.1)	Quartile 2 (284.1-397.1)	Quartile 3 (397.2-534.5)	Quartile 4 (≥534.6)	
**All-cause mortality**						
No. of cases/person-years	1192/24301	269/5154	322/6501	279/6471	322/6175	
Model 1	1.01 (0.86, 1.19)	1.00 [Reference]	0.94 (0.78, 1.14)	0.85 (0.67, 1.07)	0.99 (0.8, 1.22)	0.987
Model 2	1.05 (0.90, 1.22)	1.00 [Reference]	0.93 (0.77, 1.13)	0.90 (0.73, 1.11)	1.05 (0.85, 1.29)	0.534
Model 3	1.08 (0.92, 1.26)	1.00 [Reference]	1.01 (0.78, 1.31)	0.93 (0.71, 1.22)	1.22 (0.93, 1.61)	0.373
**Cardiovascular mortality**						
No. of cases/person-years	446/24301	95/5154	121/6501	100/6471	130/6175	
Model 1	1.09 (0.90, 1.33)	1.00 [Reference]	1.07 (0.77, 1.49)	0.94 (0.65, 1.37)	1.17 (0.87, 1.56)	0.360
Model 2	1.12 (0.92, 1.35)	1.00 [Reference]	1.01 (0.74, 1.38)	1.00 (0.71, 1.42)	1.24 (0.93, 1.65)	0.125
Model3	1.13 (0.93, 1.37)	1.00 [Reference]	1.02 (0.74, 1.39)	1.02 (0.72, 1.44)	1.26 (0.94, 1.69)	0.108

Abbreviations: CI, confidence interval; CKD, chronic kidney disease; HR, hazard ratio; Model 1: Adjusted for NHANES circles, age (continuous, year), and gender (male and female); Model 2: Further adjustment for race (Hispanic, non-Hispanic white, non-Hispanic black, and others), education (less than high school, high school and college or higher), marital status (married, separated, and never married), family poverty-to-income ratio (≤1, 1-4, ≥4), BMI (continuous, kg/m²), smoking (never, former, current), drinking (never, former, current), physical activity (low, moderate, high), diet quality (continuous, HEI-2015 score), systolic blood pressure (continuous, mmHg), eGFR(continuous, ml/min/1.73m²) based on model 1; Model 3: Further adjustment of MMA (continuous, nmol/L) based on model 2; [a] *P* values for trend were tested by using the median level of each quartile vitamin B_{12} levels and modeling it as a continuous variable in model 3.

Table S3. Subgroup analyses for the HRs of MMA and CVD mortality among U.S. adults with CKD.

Subgroup	MMA levels (nmol/L), HR (95% CI)					*P* for interaction [a]
	Log (MMA)	Quartile 1 (<123)	Quartile 2 (123-169)	Quartile 3 (170-239)	Quartile 4 (≥240)	
**Age, years**						0.819
<65	1.83 (1.14, 2.91)	1.00 [Reference]	1.35 (0.71, 2.54)	1.07 (0.52, 2.18)	2.56 (1.19, 5.52)	
≥65	1.24 (1.03, 1.50)	1.00 [Reference]	1.11 (0.75, 1.66)	1.15 (0.77, 1.69)	1.69 (1.14, 2.50)	
**Sex**						0.946
Male	1.16 (0.93, 1.46)	1.00 [Reference]	1.30 (0.84, 2.01)	1.25 (0.80, 1.95)	1.69 (1.07, 2.68)	
Female	1.70 (1.27, 2.28)	1.00 [Reference]	1.14 (0.67, 1.94)	1.16 (0.68, 1.99)	2.10 (1.22, 3.61)	
**BMI, kg/m²**						0.734
<30	1.18 (0.96, 1.46)	1.00 [Reference]	1.23 (0.81, 1.87)	1.08 (0.70, 1.68)	1.65 (1.07, 2.53)	
≥30	1.71 (1.21, 2.42)	1.00 [Reference]	1.08 (0.60, 1.92)	1.24 (0.71, 2.19)	1.96 (1.08, 3.54)	
**Smoking status**						0.689
Never or former	1.32 (1.10, 1.58)	1.00 [Reference]	1.34 (0.91, 1.96)	1.26 (0.86, 1.85)	1.90 (1.29, 2.79)	
Current	1.39 (0.81, 2.38)	1.00 [Reference]	1.07 (0.49, 2.36)	1.31 (0.58, 3.00)	2.09 (0.86, 5.09)	

**Alcohol consumption**						0.191
None	1.22 (0.92, 1.62)	1.00 [Reference]	1.29 (0.73, 2.28)	1.23 (0.69, 2.21)	1.84 (1.03, 3.29)	
Moderate	1.37 (1.08, 1.73)	1.00 [Reference]	1.18 (0.75, 1.85)	1.09 (0.69, 1.73)	1.72 (1.08, 2.75)	
Heavy	1.58 (0.55, 4.52)	1.00 [Reference]	1.20 (0.26, 5.49)	1.94 (0.42, 8.85)	3.35 (0.60, 18.58)	
**Physical activity**						0.057
Low	1.44 (1.10, 1.89)	1.00 [Reference]	1.22 (0.7, 2.15)	1.35 (0.76, 2.37)	2.26 (1.27, 4.02)	
Moderate	1.65 (1.10, 2.48)	1.00 [Reference]	1.34 (0.60, 2.98)	1.73 (0.77, 3.88)	2.57 (1.1, 5.96)	
High	1.09 (0.83, 1.43)	1.00 [Reference]	1.13 (0.68, 1.87)	0.86 (0.50, 1.46)	1.33 (0.79, 2.26)	
**eGFR, ml/min/1.73m²**						0.914
<60	1.20 (0.94, 1.54)	1.00 [Reference]	0.80 (0.41, 1.54)	0.94 (0.51, 1.72)	1.34 (0.73, 2.43)	
≥60	1.40 (1.10, 1.77)	1.00 [Reference]	1.46 (0.99, 2.15)	1.40 (0.91, 2.16)	2.10 (1.33, 3.31)	
**CKD stage**						0.930
1	1.09 (0.71, 1.68)	1.00 [Reference]	1.12 (0.60, 2.10)	0.77 (0.34, 1.73)	1.84 (0.75, 4.52)	
2	1.62 (1.16, 2.26)	1.00 [Reference]	1.88 (1.09, 3.26)	1.91 (1.06, 3.45)	2.63 (1.44, 4.79)	
Stage3-5	1.20 (0.94, 1.54)	1.00 [Reference]	0.80 (0.41, 1.54)	0.94 (0.51, 1.72)	1.34 (0.73, 2.43)	

Abbreviations: BMI, body mass index; CI, confidence interval; CKD, chronic kidney disease; eGFR, estimated glomerular filtration rate; HR, hazard ratio; Model was adjusted for NHANES circles, age (continuous, year), and sex (male and female), race (Hispanic, non-Hispanic white, non-Hispanic black, and others), education (less than high school, high school and college or higher), marital status (married, separated, and never married), family poverty-to-income ratio (≤1, 1-4, ≥4), BMI (continuous, kg/m²), smoking (never, former, current), drinking (never, former, current), physical activity (low, moderate, high), diet quality (continuous, HEI-2015 score), systolic blood pressure (continuous, mmHg), eGFR(continuous, ml/min/1.73m²), vitamin B_{12} (continuous, pmol/L); [a] *P* for interaction was estimated by including an interaction term of the composite MMA and stratifying variables in the Cox regression model and testing using the Wald test.

Table S4. HRs (95% CIs) of all-cause mortality and cardiovascular mortality according to MMA levels after excluding CKD patients with cardiovascular disease and diabetes (*n* = 1335).

Cause of death	MMA levels (nmol/L), HR (95% CI)					*P* for trend [a]
	Log (MMA)	Quartile 1 (<117.0)	Quartile 2 (117.0-159.0)	Quartile 3 (160.0-213.0)	Quartile 4 (≥214.0)	
**All-cause mortality**						
No. of cases/person-years	496/13789	54/4037	94/3569	129/2878	219/3305	
Model 1	1.36 (1.08, 1.71)	1.00 [Reference]	0.97 (0.62, 1.53)	1.36 (0.89, 2.07)	1.48 (1.02, 2.14)	<0.001
Model 2	1.44 (1.13, 1.83)	1.00 [Reference]	1.08 (0.70, 1.67)	1.61 (1.01, 2.54)	1.81 (1.25, 2.61)	<0.001
Model 3	1.44 (1.13, 1.84)	1.00 [Reference]	1.08 (0.70, 1.67)	1.61 (1.02, 2.54)	1.80 (1.25, 2.61)	<0.001
**Cardiovascular mortality**						
No. of cases/person-years	156/13789	19/4037	28/3569	34/2878	75/3305	
Model 1	1.38 (1.01, 1.88)	1.00 [Reference]	0.79 (0.46, 1.36)	1.01 (0.52, 1.99)	1.37 (0.88, 2.13)	0.032
Model 2	1.33 (0.99, 1.79)	1.00 [Reference]	0.76 (0.43, 1.36)	1.24 (0.52, 2.92)	1.39 (0.80, 2.42)	0.031
Model 3	1.33 (0.99, 1.79)	1.00 [Reference]	0.76 (0.43, 1.35)	1.24 (0.52, 2.92)	1.40 (0.80, 2.44)	0.030

Abbreviations: CI, confidence interval; CKD, chronic kidney disease; MMA, methylmalonic acid; HR, hazard ratio; Model 1: Adjusted for NHANES circles, age (continuous, year), and sex (male and female); Model 2: Further adjustment for race (Hispanic, non-Hispanic white, non-Hispanic black, and others), education (less than high school, high school and college or higher), marital status (married, separated, and never married), family poverty-to-income ratio (≤1, 1-4, ≥4), BMI (continuous, kg/m²), smoking (never, former, current), drinking (never, former, current), physical

activity (low, moderate, high), diet quality (continuous, HEI-2015 score) systolic blood pressure (continuous, mm Hg), eGFR(continuous, ml/min/1.73m²) based on model 1; Model 3: Further adjustment for vitamin B12 (continuous, pmol/L) based on model 2; [a] *P* values for trend were tested by using the median level of each quartile MMA levels and modeling it as a continuous variable.

Table S5: HRs (95% CIs) of all-cause mortality and cardiovascular mortality according to MMA levels after excluding CKD patients with less than 3 years of follow-up (*n* = 2349).

Cause of death	MMA levels (nmol/L), HR (95% CI)					*P* for trend [a]
	Log (MMA)	Quartile 1 (<121.0)	Quartile 2 (121.0-165.0)	Quartile 3 (166.0-229.0)	Quartile 4 (≥230.0)	
**All-cause mortality**						
No. of cases/person-years	952/23886	142/7458	197/5790	253/5390	360/5248	
Model 1	1.49 (1.25, 1.77)	1.00 [Reference]	1.31 (0.97, 1.76)	1.37 (0.99, 1.9)	1.95 (1.51, 2.52)	<0.001
Model 2	1.54 (1.27, 1.86)	1.00 [Reference]	1.36 (1.01, 1.84)	1.5 (1.06, 2.14)	2.13 (1.58, 2.88)	<0.001
Model 3	1.54 (1.27, 1.87)	1.00 [Reference]	1.36 (1.01, 1.84)	1.5 (1.06, 2.13)	2.13 (1.58, 2.88)	<0.001
**Cardiovascular mortality**						
No. of cases/person-years	351/23886	51/7458	80/5790	81/5390	139/5248	
Model 1	1.38 (1.13, 1.68)	1.00 [Reference]	1.36 (0.88, 2.1)	1.07 (0.71, 1.60)	1.89 (1.34, 2.67)	<0.001
Model 2	1.36 (1.10, 1.67)	1.00 [Reference]	1.41 (0.89, 2.24)	1.12 (0.71, 1.78)	1.91 (1.26, 2.88)	0.001
Model 3	1.36 (1.10, 1.68)	1.00 [Reference]	1.41 (0.89, 2.24)	1.12 (0.71, 1.78)	1.91 (1.26, 2.89)	0.001

Abbreviations: CI, confidence interval; CKD, chronic kidney disease; MMA, methylmalonic acid; HR, hazard ratio; Model 1: Adjusted for NHANES circles, age (continuous, year), and sex (male and female); Model 2: Further adjustment for race (Hispanic, non-Hispanic white, non-Hispanic black, and others), education (less than high school, high school and college or higher), marital status (married, separated, and never married), family poverty-to-income ratio (<1, 1-4, >4), BMI (continuous, kg/m²), smoking (never, former, current), drinking (never, former, current), physical activity (low, moderate, high), diet quality (continuous, HEI-2015 score) systolic blood pressure (continuous, mmHg), eGFR(continuous, mL/min/1.73m²) based on model 1; Model 3: Further adjustment for vitamin B12 (continuous, pmol/L) based on model 2; [a] *P* values for trend were tested by using the median level of each quartile MMA levels and modeling it as a continuous variable.

Table S6. HRs (95% CIs) of all-cause mortality according to MMA levels among CKD patients with further adjustment for several biomarkers.

Cause of death		MMA levels (nmol/L), HR (95% CI)					*P* for trend [a]
		Log (MMA)	Quartile 1	Quartile 2	Quartile 3	Quartile 4	
**All-cause mortality**							
No. of cases/person-years		1192/24301	169/7670	233/5888	337/5851	453/4892	
Model 1	2589	1.09 (0.90, 1.34)	1.00 [Reference]	1.20 (0.91, 1.57)	1.28 (0.94, 1.76)	2.01 (1.54, 2.62)	<0.001
Model 2	1427	1.36 (1.10, 1.69)	1.00 [Reference]	1.29 (0.91, 1.84)	1.29 (0.90, 1.83)	1.77 (1.30, 2.41)	<0.001

Model 3	2587	1.49 (1.28, 1.73)	1.00 [Reference]	1.16 (0.89, 1.51)	1.23 (0.90, 1.69)	1.91 (1.47, 2.48)	<0.001
Model 4	2589	1.51 (1.29, 1.76)	1.00 [Reference]	1.20 (0.92, 1.57)	1.30 (0.95, 1.77)	2.02 (1.55, 2.63)	<0.001
Cardiovascular mortality							
No. of cases/person-years		446/24301	61/7670	95/5888	115/5851	175/4892	
Model 1	2589	1.30 (1.08, 1.57)	1.00 [Reference]	1.29 (0.85, 1.97)	1.04 (0.69, 1.58)	1.76 (1.18, 2.63)	0.088
Model 2	1427	1.17 (0.90, 1.5)	1.00 [Reference]	1.17 (0.72, 1.90)	1.10 (0.72, 1.69)	1.41 (0.93, 2.14)	0.095
Model 3	2587	1.27 (1.05, 1.53)	1.00 [Reference]	1.24 (0.83, 1.85)	0.99 (0.66, 1.48)	1.63 (1.12, 2.39)	0.001
Model 4	2589	1.30 (1.08, 1.57)	1.00 [Reference]	1.30 (0.85, 1.99)	1.05 (0.69, 1.60)	1.77 (1.18, 2.66)	<0.001

Abbreviations: CI, confidence interval, CKD, chronic kidney disease; MMA, methylmalonic acid; HR, hazard ratio; Model 1: Adjusted for NHANES circles, age (continuous, year), and sex (male and female), race (Hispanic, non-Hispanic white, non-Hispanic black, and others), education (less than high school, high school and college or higher), marital status (married, separated, and never married), family poverty-to-income ratio (<1, 1-4, >4), BMI (continuous, kg/m^2), smoking (never, former, current), drinking (never, former, current), physical activity (low, moderate, high), diet quality (continuous, HEI-2015 score), systolic blood pressure (continuous, mm Hg), eGFR(continuous, ml/min/1.73m^2), vitamin B_{12} (continuous, pmol/L); Model 2: model 1+ homocysteine; Model 3: model 1+HbA_{1c}; Model 4: model 1+total cholesterol; [a] *P* values for trend were tested by using the median level of each quartile MMA levels and modeling it as a continuous variable.
